# The relationship between staff skill mix, costs and outcomes in intermediate care services

**DOI:** 10.1186/1472-6963-10-221

**Published:** 2010-07-29

**Authors:** Simon Dixon, Billingsley Kaambwa, Susan Nancarrow, Graham P Martin, Stirling Bryan

**Affiliations:** 1 School of Health and Related Research, University of Sheffield, Regent Court, 30 Regent Street, Sheffield S1 4DA, UK; 2 Health Economics Unit, Health and Population Sciences, University of Birmingham, Edgbaston, Birmingham B15 2TT, UK; 3 Centre for Health and Social Care Research, Sheffield Hallam University, Montgomery House, 32 Collegiate Crescent, Collegiate Campus, Sheffield S10 2BP, UK; 4 Department of Health Sciences, University of Leicester, Adrian Building, University Road, Leicester LE1 7RH, UK; 5 Centre for Clinical Epidemiology and Evaluation, University of British Columbia, Seventh Floor, 828 West 10th Avenue, Research Pavilion, Vancouver BC V5Z 1M9, Canada

## Abstract

**Background:**

The purpose of this study was to assess the relationship between skill mix, patient outcomes, length of stay and service costs in older peoples' intermediate care services in England.

**Methods:**

We undertook multivariate analysis of data collected as part of the National Evaluation of Intermediate Care Services. Data were analysed on between 337 and 403 older people admitted to 14 different intermediate care teams. Independent variables were the numbers of different types of staff within a team and the ratio of support staff to professionally qualified staff within teams. Outcome measures include the Barthel index, EQ-5D, length of service provision and costs of care.

**Results:**

Increased skill mix (raising the number of different types of staff by one) is associated with a 17% reduction in service costs (p = 0.011). There is weak evidence (p = 0.090) that a higher ratio of support staff to qualified staff leads to greater improvements in EQ-5D scores of patients.

**Conclusions:**

This study provides limited evidence on the relationship between multidisciplinary skill mix and outcomes in intermediate care services.

## Background

There has been growing international interest in 'workforce engineering and redesign' over recent years, which has resulted in an increase in research exploring the impact of different approaches to staffing on patient and service outcomes, particularly in the areas of medicine and nursing. There are several drivers for workforce change including skills shortages; productivity improvements; cost containment; quality improvement; technological innovation; and health sector reform. The modernisation of the National Health Service has led to substantial changes to the numbers and types of staff, and their ways of working. For instance, workforce shortages and restructuring in the UK have created opportunities for staff to perform roles that are outside their traditional scope of practice[1].

Intermediate care (IC) is a valuable setting in which to explore new ways of working. Many IC services operate at the interface of numerous agencies, settings and professional groups, and require workforce structures that can reflect and respond to this complexity [2]. IC services tend to have non-hierarchical management structures; and staff are often supervised by someone whose professional background is different to their own. Medical practitioners are sometimes the 'gatekeeper' to IC, however their level and mode of involvement varies [3]and non-medical practitioners often have a great deal of autonomy [2]. Finally, IC services can be delivered in a variety of locations, including the patients' own home, nursing homes, hospitals and community centres.

Following the National Service Framework for Older People (31), the number and type of community based services for older people have grown substantially and are set to expand further as acute care services are progressively moved to primary and community care settings. Intermediate care services have diverse models of staffing, however typically intermediate care teams are multidisciplinary [4-14] even in usual care settings, or when labelled 'nurse led unit', or 'GP led unit'. They are likely to include input from physiotherapy, occupational therapy and therapy assistants [5,10]. A wide range of other staff may be involved in the delivery of intermediate care, however this varies greatly across the different services [13]. There is no evidence about the 'best way' to staff an intermediate care service, and this is likely to depend on the setting and purpose of the service[10]. Comparable studies are difficult to find, as most workforce studies explore the relationship between two different practitioners rather than multidisciplinary arrangements.

Only one experimental study specifically examined the impact of different models of staffing on costs and outcomes [8] by comparing hospital at home with care on a hospital ward. Staffing models were not attributed to outcomes, however the research showed that cost efficiency of services was negatively influenced by employing high grade nurses in roles with little direct clinical input. In contrast, the costs of the other members of the multidisciplinary team (eg therapists) constituted a relatively small component of the total cost. The authors suggested that increasing the proportion of nurses involved in more direct nursing care could reduce the costs of the service.

There is evidence from a number of qualitative studies that intermediate care requires staff to work across professional boundaries, and that initially, this can create tensions, however generally this improves with time, and is perceived by staff to enhance patient and service outcomes[9,15,16].

The literature demonstrates that patient satisfaction is positively associated with well trained workers and respectful staff, however is negatively associated with poor recruitment and retention and delayed or absent workers [17]. It is also evident that service user perceptions of service quality are likely to be positively influenced by patient characteristics, such as age, and organisational characteristics such as the intensity of care received, staffing organisation, employment conditions for staff, good recruitment and retention rates and greater levels of staff experience and training [18]. Many of the same factors have been found to significantly influence patient functional gain [19]. Staff experience and training such as competency of support workers in delivering rehabilitation and the presence of advanced practice nurses in teams can improve patient functional gains. Similarly patient functional outcomes can also be enhanced by greater intensity of care, greater therapy and general staffing levels and the use of agency staff have also been found to improve functional gains and outcomes.

Teamwork, team order and organisation have also been found to improve functional outcomes [20]. Several studies however have indicated that there are other factors that contribute to functional gain outside of these workforce variables. Patient characteristics such as higher cognitive ability of patients [21], the patient mix [19] and a longer stay in a post-acute care facility [21] were all found to positively impact on functional gain.

A systematic review of the 'Evidence for the effectiveness of intermediate care' [22] found that the evidence supporting the development of specific intermediate care services is quite heterogeneous, and still lacking. They reported that overall, intermediate care services are not associated with adverse consequences for recipients, and intensive therapy can improve physical outcomes and patient satisfaction. Extrapolating from the main study findings, it appears that despite large variations in staffing across services, there is little measurable effect on the outcomes for service users. These findings suggest that there may be potential for efficiency savings in intermediate care services through the identification of more effective models of interprofessional team organisation.

There is a need for greater understanding and consultation around service user preferences for different types of staffing (type, roles, numbers etc). For instance, Brown et al [23] found that home care workers were the most valued service provider in the health and social care team and it did not matter to the service user whether or not the team was integrated as long as their needs were met.

The aim of this study is to assess the relationship between skill mix, patient outcome, length of stay and cost. This was part of a larger study exploring the relationship between staffing and patient outcomes[24], and involved the reanalysis of data from a National Evaluation of Intermediate Care with the addition of data relating to the skill mix of the teams included within the study.

## Methods

The National Evaluation of Intermediate Care Services [25] was undertaken by the Universities of Birmingham and Leicester. It involved extensive qualitative and quantitative data collection within five case-study sites in England between January 2003 and November 2004. The processes used for the collection and analysis of quantitative data in the case-study sites are described in detail elsewhere [25,26].

The case-studies were five primary care trusts selected as to represent 'whole systems' (an area with a specific geographical boundary) of intermediate care. By studying whole systems as opposed to individual service models we aimed to achieve a more detailed understanding of the implementation of intermediate care and its impact upon system-level costs and outcomes.

Quantitative data were collected by staff employed by the intermediate care services according to protocols established by the evaluation team. Staff completed a study proforma with their patients, at the point of entry to the service, and then further questions were completed on the day of discharge, transfer or following the end of service provision. All intermediate care admissions over a defined period were included.

Data were available on patient age, gender, Barthel score at admission and discharge, EQ-5D at admission and discharge, type of service defined in terms of admission avoidance or other, and location of service in terms of residential or non-residential.

The Barthel score is a measure of a patient's ability to undertake a set of activities of daily living, such as feeding, bathing and grooming. It is typically completed by the health professional, and is scored on a scale of zero to twenty with zero indicating that the patient is fully dependent on others for all activities, and twenty indicating that the patient is independent [27,28]. The EQ-5D, formerly know as the EuroQol, is a generic measure used primarily by economists to calculate quality adjusted life years (QALYs). It uses a single question to assess each of five health domains; mobility, self-care, usual activities, pain/discomfort and anxiety/depresssion. The EQ-5D has a complex scoring system, which ranges from 1 which indicates full health, through to -0.59 [29].

Data on skill mix were collected as descriptive data, but not included in any of the analyses undertaken to date. These data recorded the types of health care worker included in each of the teams at the time of the evaluation, and the number of whole-time equivalents. These were summarised in terms of two skill mix variables; ratio of support workers to qualified staff and the number of different professions included within the team. For the purposes of these two measures, support workers included staff involved in the direct delivery of patient care but who do not have a professional qualification, and included assistant practitioners, therapy assistants, support workers, generic rehabilitation assistants, health care assistants and social care workers. Staff were categorised as 'qualified' if they had a recognisable professional title which is associated with tertiary training, and included nurses, doctors, allied health practitioners and social workers. The 'number of different types of professions' was simply a count of the numbers of different types of practitioners (including support workers) involved in the delivery of patient care. Additionally, the team data were used to calculate the total number of WTEs employed, as a proxy for the size of the service.

NHS ethical approval for the secondary analysis was obtained in 2006 (06/Q1606/132).

## Analyses

Data used in the National Evaluation, plus the additional variables defined from the team data were used to undertake a set of multivariate analyses. These were to assess:

▪ The impact of skill mix on outcomes of care as measured by the change in the Barthel index.

▪ The impact of skill mix on outcomes of care as measured by the change in the EQ-5D.

▪ The impact of skill mix on length of care episode (or length of service provision).

▪ The impact of skill mix on costs of care as measured.

Based on previous analyses of costs and outcomes, the relationship with age was thought to be monotonic but non-linear, and so age-squared was used as an independent variable. Likewise, based on economic theory, for the analysis of costs total WTE squared was also defined to help identify possible economies of scale across the teams.

Multivariate analyses were undertaken using individual patient data, but taking into account the clustering of cases within teams within STATA. Ordinary Least Squares (OLS) regression was undertaken for the analyses of outcomes (change in EQ-5D and Barthel) as dependent variables, whilst generalised linear models with a log link and gamma distribution were used for the analyses of length of stay and cost per patient. Generalised linear models (GLMs) are considered to be more appropriate for the analysis of skewed and heteroscedastic data while retaining the original scale of the data [30]. To aid interpretation of GLM coefficients, the exponents of the coefficients were calculated. These can be interpreted as the proportional change of the dependent variable because of a change of one unit in the independent variable (32).

When interpreting the statistical significance of the models, we have adopted the approach of Bland [31] whereby p-values greater than 0.10 indicate little or no evidence of a relationship, values between 0.05 and 0.10 indicate weak evidence of a relationship, values between 0.01 and 0.05 indicate evidence of a difference or relationship and values less than 0.01 indicate strong evidence of a difference or relationship.

Additionally, the specification of the estimated regression equations was assessed using the Ramsey REST test [32]. This test performs auxiliary regressions that add in powers of the fitted values to the original equations. Statistically significant coefficients on these new terms have been found to be indicative of misspecification.

## Results

Across the four analyses, data were available on between 337 and 403 patients, describing costs and outcomes across 14 separate teams. Patient and team characteristics are summarised in Table 1.

**Table 1:** Description of patient and team characteristics

Patient characteristics	Median	Min;Max
Age	82.14	62.34;100.63

Baseline Barthel	15.00	3.00;20.00

Change in Barthel (n = 398)	1.00	-5.00;14.00

Baseline EQ5D	0.52	-0.59; 1.00

Change in EQ5 D (n = 349)	0.07	-1.11; 1.16

Length of care (days)	31.00	1.00; 232.00

Cost per patient (£)	1241.68	40.07; 15,323.60

Gender - n (%) for females	299 (74.19)	

Team characteristics	Median	Min-Max

Ratio of support staff to qualified staff	0.67	0.00; 4.00

Number of different types of staff	5.00	3.00; 9.00

Total number of staff (WTEs)	7.75	1.82; 23.70

IC function - n (%) for acute admission avoidance	215 (53.35)	

IC setting - n (%) for residential IC	102 (25.31)	

**Note: **n = 403 unless otherwise stated. 403 observations were used as this sample represents the set of patients on which all four sets of regression analyses were run.

### The relationship between skill mix and outcomes of care as measured by the Barthel index

There is strong evidence that less independent patients on admission (as indicated by lower Barthel scores) were associated with greater improvements in Barthel over the period of care (Table 2). In particular, for each one unit decrease in the baseline Barthel score, the change in Barthel score increased by 0.2854. None of the skills staffing parameters were statistically significant. Whilst the overall explanatory power the relationship was significant, as evidenced by the block F-test, there was also evidence of possible misspecification.

**Table 2:** Regression results

	Change in Barthel score^1^ n = 398	Change in EQ5D score^2^ n = 337	Length of care (days) n = 403	Cost (£s) n = 403
Age	0.3085 (0.2272)	0.0336 (0.0366)	-0.0490 (0.0890)	0.1273*** (0.0363)
Age squared	-0.0022 (0.0014)	-0.0002 (0.0002)	-0.0002 (0.0005)	-0.0008*** (0.0002)
Gender	0.2852 (0.2469)	0.0670 (0.0365)	-0.0225 (0.0754)	0.0352 (0.0379)
Baseline Barthel score	-0.2854*** (0.0794)		-0.0136 (0.0111)	0.0039 (0.0083)
Baseline EQ5 D score		-0.4363*** (0.0651)	0.1067 (0.1026)	0.0450 (0.1075)
Admission avoidance	0.5030 (0.3478)	0.0044 (0.0400)	-0.2000** (0.0832)	0.0565 (0.0618)
Residential care	0.6126 (0.6976)	0.0582* (0.0327)	0.0835 (0.2587)	1.5892*** (0.3578)
Length of care	-0.0003 (0.0057)	-0.0003 (0.0005)	-	0.0257*** (0.0030)
Ratio of support to qualified staff	0.2277 (0.4819)	0.0464* (0.0254)	-0.0564 (0.1042)	0.0600 (0.1076)
Number of different staff types	-0.0529 (0.1532)	0.0161 (0.0103)	0.0470 (0.0608)	-0.1827** (0.0715)
Total number of staff	-0.0064 (0.0258)	-0.0005 (0.0011)	0.0010 (0.0104)	0.2026*** (0.0646)
Total number of staff squared				-0.0085*** (0.0022)
Constant	-4.9479 (8.8322)	1.1658 (1.4717)	6.0765 (3.7505)	0.8401 (1.535)

R-squared	0.1932	0.2505	0.0318	0.2730
Block F-test^3^	<0.0001	<0.0001	-	-
RESET test	0.0002	0.0441	-	-

1 Positive changes reflect gains in a patient's level of independence.

2 Positive changes reflect improvements in a patient's health related quality of life.

3 Tests the hypothesis that all parameters are equal to zero.

* 0.10 > p-value > 0.05

** 0.05 > p-value > 0.01

*** p-value <0.01

### The relationship between skill mix and outcomes of care as measured by the EQ-5D

There is strong evidence that lower EQ-5D scores on admission are associated with greater improvements in EQ-5D over the period of care (Table 2). For each one unit decrease in the baseline EQ-5D score, the change in EQ-5D score increased by 0.4363. There is also weak evidence that residential intermediate care services, and higher support staff to qualified staff ratios are associated with greater improvements in EQ-5D scores. The gain in EQ-5D for individuals in residential care was 0.0582 units bigger than that of individuals in non-residential care while a 1 unit increase in the ratio of support staff to qualified staff increased the change in EQ-5D by 0.0464 units. Overall, the relationship has significant explanatory power, but misspecification is suggested.

### The relationship between skill mix and process of care as measured by length of care episode

Acute admission avoidance schemes are strongly associated with having shorter periods of intermediate care: the length of care for individuals in such schemes was about 18% shorter [exp(-0.2000) = 0.8187] compared to that of individuals in other schemes. None of the skills staffing parameters were statistically significant.

### The relationship between skill mix and costs of care

There is strong evidence that older patients were associated with higher costs but these costs begin to fall as patients become more elderly. For each one year increase in age, costs per case rose by 13.58% [exp(0.1273) = 1.1358] and further analysis indicates that costs begin to fall when individuals reach around 80 years old. Residential services and longer periods of care were strongly associated with higher costs. Costs for residential services were almost five times bigger than those for non-residential services [exp(1.5892) = 4.8998] while an increase of 1 day in the length of care was associated with a 2.60% increase in costs [exp(0.0257) = 1.0260). There was evidence that greater numbers of different types of staff were associated with lower costs (Table 2). Having an extra category of staff decreased costs by about 17% [exp(-0.1827 = 0.8330]. The coefficients on total staff numbers and total staff numbers squared suggest that cost per case initially increase by 22.46% [exp(0.2026) = 1.2246] as teams grow by a factor of one individual, but after then begin to fall. Further analysis indicates that the point at which cost per case begins to fall is around 12 WTE staff which is 3 WTE staff members larger than the largest team in the study as shown in Table 1.

## Discussion and conclusions

The analyses show that costs and outcomes of intermediate care are partly explained by differences in patient and service characteristics, however, the impact of service skill mix is limited (Table 2). There is weak evidence (p = 0.090) that the ratio of support staff to qualified staff impact on health gains (measured by the change in EQ-5D) seen during care, with higher proportions of support staff being associated with greater improvement. There is stronger evidence (p = 0.011) that higher numbers of different types of staff are associated with lower costs.

There are several possible explanations for the greater improvements in EQ-5D in patients when who utilise more support staff (SS) relative to qualified staff (QS). Qualitative feedback from the same study suggests that support staff spend more time with patients than qualified staff, and perform more of the 'hands on' work, which may lead to better improvements in outcome. Alternatively, it could mean that additional SS allow a better service to be delivered, for example, increasing the number of SS staff may allow for service development. This second interpretation is in line with findings seen in general practice [33].

This second interpretation is less plausible as some aspects of service expansion will be controlled for by the 'total number of staff' variable within the regression. In other words, increasing SS staff without reducing QS staff is not responsible for the better outcomes associated with the higher support staff to qualified staff ratios.

Other possible explanations are that intermediate care patients may not require the intensive or specialised treatment of support staff, thus a higher ratio of SS to QS may be the optimum combination that will lead to better outcomes. Similarly, it may be that those patients who do require more specialised input are directed to services that provide that input.

The impact of greater numbers of different types of staff on costs could reflect economies produced by specialisation. Understanding how costs were calculated within the National Evaluation is important before considering this issue further. Cost per patient was calculated based on a cost per day for the entire service based on budgets and an individual patient's length of care. So, cost per patient is driven either by the service budget or length of stay. As the relationship between number of different types of staff and length of care is small and statistically insignificant, it appears that the effect is through the size of the service budget. The mechanism by which service budgets are reduced is open to speculation. Two possible processes are reduced number of visits and/or the use of smaller numbers of staff.

The results also show a potential conflict between patient outcomes and costs; increasing support staff numbers relative to qualified staff appears to improve health outcomes (as measured by the EQ-5D), but if this is achieved at the expense of multidisciplinarity (as measured by numbers of different types of staff) then costs will increase.

This study is important because it uses existing data to explore the potential for a relationship between different staffing models and patient outcomes in services that are an important showcase of the NHS modernisation agenda with the introduction of new roles, integrated health and social care services, and interdisciplinary working. Accurate data on staffing and patient outcomes in community based services can be costly and difficult to capture. The relationships demonstrated within this study indicate that at the very least, further research is warranted into the relationship between outcomes and staffing to support more efficient and effective ways to deliver patient care.

### Limitations

The regressions have reasonable explanatory power, however there is evidence from the RESET test that there is misspecification. Possible causes of this could be the choice of regression technique or the omission of relevant variables. The Barthel and EQ-5D scores to possess some characteristics that are similar to truncated data, with minimum and maximum permitted scores (and hence changes in scores). Consequently, some studies that have analysed quality of life data of this kind have used truncated regressions and censored least absolute deviations (CLAD) regressions [34,35]. These were undertaken, however, they did not affect the results appreciably.

Likewise, the possibility of omitted variables was investigated by analysing other specifications that included interaction terms between the staff mix variables. These additional regressions led to problems with interpretation probably caused by using so many cluster-based independent variables in the face of so few clusters. The RESET tests also indicated that misspecification problems persist even the presence of these more complex specifications.

Two individuals recorded gains in EQ-5D of 1.59, implying that they moved from the worst possible state at admission to the best state at discharge. These 'extreme' changes might reflect misunderstanding on the part of the respondents. Excluding these two individuals from the analysis however did not alter the results above.

Whilst the presence of clustering was taken into account in the analysis, it should be noted that the small number of clusters will limit our ability to detect any associations that are present. This is exacerbated by the limited variability seen between the clusters in terms of skill mix (Table 1).

Interpretation of the results is also limited by the fact that we do not know the number of visits and type of therapy/care provided at the visits. So, for example, we do not know whether the improved outcomes associated with support staff is due to the type of input ('x' rather than 'y') or more frequent input ('more of x').

It is feasible that the relationship between staffing numbers and outcomes is due to the staff identifying patients with greater potential to improve and allocating more staffing resources to those patients. However, if this were the case, the mechanisms by which this was performed was not clear or systematic.

In conclusion, this study provides limited evidence of the role of skill mix on the costs and outcomes of intermediate care services. The work is based around an observational dataset and the use of skill mix variables at the service level, which together may limit our ability to identify possible relationships. A controlled study with clearly defined packages of inputs being provided to patients, would provide a clearer picture of how skill mix can impact on costs and outcome of intermediate care services. Until such work is done, services will continue to develop in a largely piecemeal way, with the consequences of this being largely hidden.

## Competing interests

The authors declare that they have no competing interests.

## Authors' contributions

SN and GPM helped link staffing to other quantitative data from the national evaluation. SB, SD and BK developed the analysis plan for the econometric analysis. SB and BK performed the econometric analysis, and interpreted and discussed the results. SD, SN and GPM wrote the first draft of the manuscript. All authors critically revised the manuscript, and read and approved the final manuscript. SN obtained the NIHR funding for the project.

## Pre-publication history

The pre-publication history for this paper can be accessed here:

http://www.biomedcentral.com/1472-6963/10/221/prepub
